# Essential Safety Considerations for Total Hip Arthroplasty: Pelvic and Spine Alignment Across Age Groups in Women at an Osteoporosis Outpatient Clinic—A Retrospective Observational Study

**DOI:** 10.3390/jcm14061847

**Published:** 2025-03-09

**Authors:** Makoto Shirono, Norio Imai, Daisuke Homma, Yuki Hirano, Yoji Horigome, Hiroyuki Kawashima

**Affiliations:** 1 Division of Orthopedic Surgery, Department of Regenerative and Transplant Medicine, Niigata University Graduate School of Medical and Dental Sciences, 1-757, Asahimachi-do-ri, Chuou ku, Niigata City 951-8510, Japan; mshiro54johney@yahoo.co.jp (M.S.);; 2 Division of Comprehensive Musculoskeletal Medicine, Niigata University Graduate School of Medical and Dental Sciences, 1-757, Asahimachi-do-ri, Chuou ku, Niigata City 951-8510, Japan

**Keywords:** spinal sagittal alignment, pelvic alignment, pelvic incidence, total hip arthroplasty

## Abstract

**Background/Objectives**: Pelvic incidence (PI) is deeply related to spinal sagittal alignment. Previous reports have demonstrated a deep association between PI and anatomical sacral slope (a-SS), underscoring the utility of a-SS in estimating PI. The investigation of temporal changes in pelvic and spinal alignment in healthy individuals is crucial for conducting surgical interventions such as total hip arthroplasty; however, these changes remain undocumented. There have been a few Japanese reports on this topic. This study explores the relationship between aging-related changes and pelvic and spinal sagittal alignment. **Methods**: By employing the methodology from a study by Imai et al., we analyzed the anterior pelvic plane (APPA), PI, pelvic tilt (PT), sacral slope (SS), a-SS, anatomical pelvic tilt (a-PT), thoracic kyphosis angle (TK), and lumbar kyphosis angle (LL), to determine the degree of kyphosis in healthy individuals. **Results**: APPA decreased over time, SS altered gradually, and PT underwent more pronounced variations with age; however, PI did not change significantly. a-SS changed early and was lower in the younger group than in the older group. Moreover, a-PT decreased with age. Spinal sagittal alignment was similar between the younger and older groups, changing gradually in LL and earlier in TK than in LL. **Conclusions**: Pelvic changes are compensated for by the pelvis, and TK changes, i.e., spinal alignment changes, are compensated for by the LL. The posterior pelvic tilt progresses with age, moving from compensation at the sacroiliac joint to compensation at the sacrum.

## 1. Introduction

In recent years, the age at which total hip arthroplasty (THA) is performed has been decreasing. It is also known that pelvis and spine sagittal alignment changes with age. There is a concern that these changes may affect THA performed at a younger age. Many young patients have a high desire for activity, and Streck et al. [1] reported predictors of activity after THA surgery. However, spinal and pelvic alignment were not included in the predictors.

Pelvic and spinal sagittal alignment is an important factor in the diagnosis and treatment of hip joint diseases, as well as spinal diseases. In recent years, sagittal alignment has been reported to be a factor in postoperative outcomes in spinal deformity, degenerative spinal disease, and THA [2,3,4]. Haffer et al. [2] suggested that THA affects spinal sagittal alignment and pelvic alignment. The significance of risk factors for complications, such as posterior impingement and anterior dislocation of the THA implant, may be increased in patients with poor alignment, and surgeons should consider spinal and pelvic alignment because of the irregular spinal sagittal alignment in post-THA patients.

As Morimoto et al. [5] reported, pelvic incidence (PI) is closely related to spinal sagittal alignment; moreover, Ike et al. [4] showed that PI is an important predictor of the risk of postoperative THA impingement and deviation from the functional safe zone in cup placement. However, Imai et al. [6] found a deep association between PI and anatomical sacral slope (a-SS), which is useful for estimating PI. Buckland and Vigdorchik [7] also examined the PI-LL mismatch effect of THA on pelvic tilt and reported an increase in posterior pelvic tilt in patients with mismatched PI-LL. It is anticipated that the incidence of THA will increase among highly active young individuals in the near future. The purpose of this study was to explore changes in the sagittal alignment of the spine and pelvis by age group to help reduce the complications associated with THA.

## 2. Materials and Methods

### 2.1. Study Design/Patient Population

The study included patients who visited our osteoporosis outpatient clinic (Shibata Hospital-Niigata Prefectural Hospital, Shibata City) between April 2015 and March 2017. (1) The patients were those who underwent standing radiographs of the thoracic and lumbar spine to determine the presence of asymptomatic spinal fractures and confirm sagittal plane alignment, (2) patients with obvious vertebral tears on thoracic and lumbar spine radiographs, and (3) those who underwent THA. Patients who had undergone hip surgery, such as total hip replacement, were excluded. The study included 245 Japanese women with a mean age of 64.3 (standard deviation [SD] 8.1; 47–84) years. In a survey conducted at an outpatient clinic for osteoporosis, which affects more women than men, all patients who met the survey’s conditions were women. Radiographs of the thoracic and lumbar spine in the standing position, as well as frontal and lateral pelvic views, were retrospectively examined for these patients. To examine intraobserver reliability, measurements were taken twice, at least 1 week apart, by the same examiner (MS) with more than 10 years of orthopedic surgical experience. To investigate interobserver reliability, the intraclass correlation coefficient was evaluated by another examiner (NI), who also had more than 10 years of experience. This study was approved by the Ethics Review Committee of the Niigata University Graduate School of Medical and Dental Sciences (No. 2017-0178), and the need for informed consent was waived due to the retrospective study nature. Strengthening the reporting of observational studies in epidemiology guidelines was referred to in the preparation of this paper.

### 2.2. Measurement of Pelvic and Spinal Parameters

**Figure 1:**
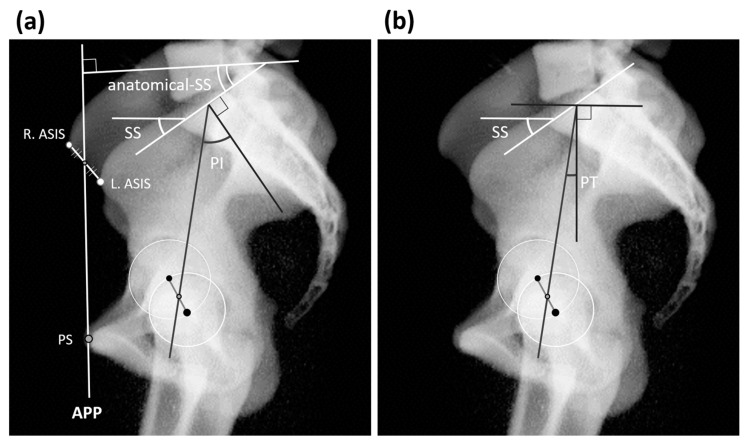
(**a**) Parameters of the pelvis. Radiograph was taken with the patient in a standing position. Sacral slope (SS) is the angle formed between the sacral plate line and the horizontal line perpendicular to the direction of the force of gravity. Anatomical SS (a-SS) is the angle formed between the S1 superior end plate and the line perpendicular to the anterior pelvic plane, which is defined as the line connecting the center point of the bilateral anterior superior iliac spine (ASIS) to the pubic symphysis. Left anterior iliac spine (L-ASIS). Right anterior superior iliac spine (R-ASIS). Anterior pelvic plane (APP). (**b**) Pelvic tilt (PT) is the angle formed between a line connecting the center point of the sacral plate to the femoral head axis line and a vertical line parallel to the direction of the force of gravity. Pelvic incidence (PI) is SS+PT.

**Figure 2:**
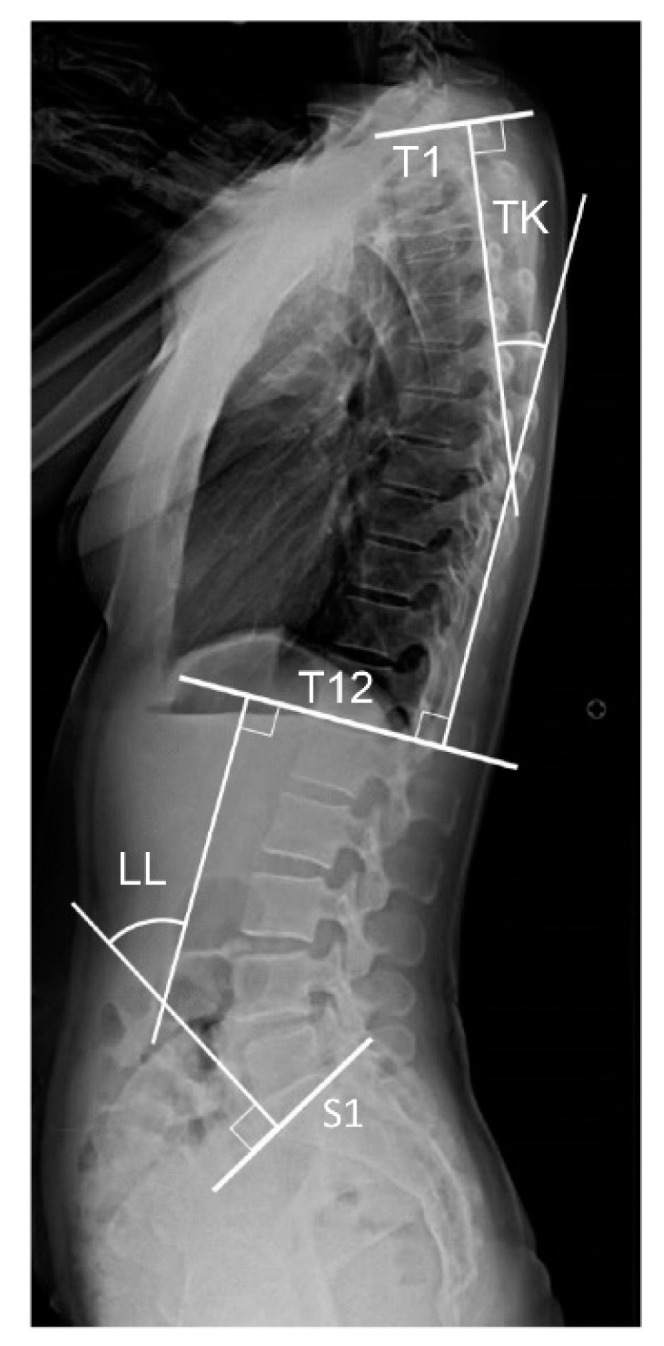
Parameters of the sagittal spine. Patient was in a standing position. Lumber lordosis (LL) is the angle formed between T12 inferior end plate and S1 superior end plate. Thoracic kyphosis (TK) is the angle formed between T1 superior end plate and T2 inferior end plate.

Thoracolumbar spinal sagittal alignment and pelvic parameters are typically evaluated on two-dimensional (2D) sagittal standing radiographs. Sacral slope (SS) is the angle formed between the sacral plate line and the horizontal line perpendicular to the direction of the force of gravity. a-SS is the angle formed between the S1 superior end plate and the line perpendicular to the anterior pelvic plane, which is defined as the line connecting the center point of the bilateral anterior superior iliac spine (ASIS) to the pubic symphysis. We measured the angles using the methods reported by Imai et al. [6,8,9]. a-SS measurement is not dependent on the femoral head and the S1 upper edge (Figure 1a). Pelvic tilt (PT) is the angle formed between the line connecting the center point of the sacral plate to the femoral head axis and the vertical line parallel to the direction of the force of gravity (Figure 1b). PI is SS+PT; SS and PT are considered positional parameters. These angles depend on horizontal or vertical lines that are perpendicular or parallel to the force of gravity and are affected by the position of the individual. Conversely, the PI angle is considered an anatomical parameter and remains the same regardless of the position of the individual. Thoracic kyphosis (TK) is the angle formed between the T1 upper endplate and the T12 endplate (Figure 2). To examine intraobserver reliability, measurements were taken twice by the same examiner (MS) at intervals of at least 1 week. To investigate interobserver reliability, the intraclass correlation coefficient was assessed by another examiner (NI).

Patients were classified based on age. Group 1 comprised patients aged 50 years or younger, Group 2 comprised patients aged 50 to 59 years, Group 3 comprised patients aged 60 to 69 years, and Group 4 comprised patients aged 70 years or older.

### 2.3. Statistical Analyses

For one-way ANOVA, we conducted a post hoc analysis for statistical power (type II (β) error) with 0.25 as the effect size (d) and 0.05 as the type I (β) error.

## 3. Results

**Figure 3:**
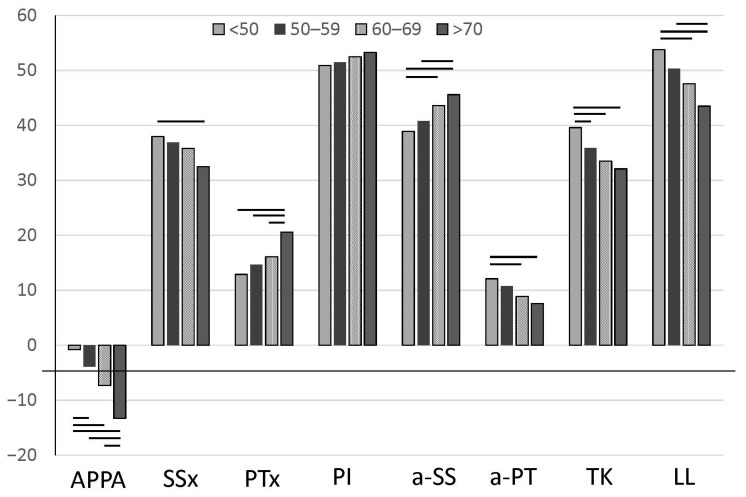
Change in various parameters by age group.

Detailed measurement values of the parameters are shown in Table 1. Changes in the values of each parameter by group are shown in Figure 3. The change in APPA decreased over time, while the change in SS was gradual, with significant differences observed only between Groups 1 and 4. PI showed no significant change. a-SS showed a change from Groups 1 and 2 to Groups 3 and 4, and a-PT showed a change from Group 1 to Groups 3 and 4. Concerning sagittal spine alignment, LL showed significant changes from Group 1 to Groups 3 and 4 and from Groups 2 to 4, and TK showed significant changes from Group 1 to Groups 2, 3, and 4. APPA decreased significantly with age starting from Group 1, i.e., backward sloping with age was observed. a-SS showed a significant difference between Groups 1, 3, and 4 and between Groups 2 and 4. a-PT showed a significant difference between Group 1 and Groups 3 and 4 and between Groups 2 and 4. There was a significant difference in LL between Groups 1, 3, and 4 and between Groups 2 and 4; a significant difference in a-SS between Groups 1, 3 and 4; a significant difference in PT between Groups 1, 3, and 4; and a significant difference in TK between Group 1 and Groups 2, 3, and 4 (Figure 3). Sacral changes were slower than the progression of pelvic retroversion. This suggests that pelvic compensation may be performed at the sacroiliac joint. The power of the one-way ANOVA was determined to be 0.981.

## 4. Discussion

**Figure 4:**
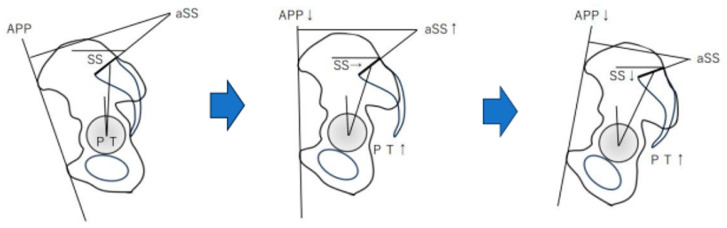
Predicted pelvic change over time: APP decreases over time, and a-SS increases. At a young age, from a to b, the sacrum does not move much, and the sacroiliac joint compensates for the sacrum to a large extent. With aging, the sacrum also tilts backward, and at age c, compensation by the spine becomes marked.

In younger patients undergoing THA, it is necessary to pay attention to the changes in the sagittal alignment of the spine and pelvis as the patients age. This study suggests that changes in the sagittal alignment of the pelvis and spine are not related to the pelvis and spine in younger individuals; however, in older individuals, the sacrum tilts backward and compensates for the loss of TK, affecting the spine and possibly compensating for it with changes in the lumbar and thoracic vertebrae. The sacral changes were slower than the progression of the pelvic retroversion. This suggests that pelvic compensation may take place at the sacroiliac joint. The difference in SS in Groups 1 and 4 of this study suggests that the sacrum may move and compensate only when the sacroiliac joints reach their limit of compensation over time. Probably, changes in the pelvis are compensated by the pelvis, and changes in TK, i.e., changes in spinal alignment, are compensated by the LL. The fact that there was no significant difference in a-SS between Groups 3 and 4 (Figure 4) suggests that the backward tilting of the pelvis continues with age, and that there is a shift from compensation at the sacroiliac joint to compensation at the sacrum with aging.

The values of these pelvic and spinal parameters have been reported to vary by country and race. As for PI, among non-Asians, Legaye and Duval-Beaupère [10] reported angles of 53° for men and 48° for women among healthy adults in Belgium and France; Vialle et al. [11] reported angles of 54° for adults in France; and Mac-Thiong et al. [12] reported angles of 53° for adults in Canada. Among Asians, Lee et al. [13] reported an angle of 48° in Koreans, Zhu et al. [14] reported a 44° angle in Koreans, and Yeh [15] reported a 49° angle in Taiwanese people, with no difference between age groups or sex. In Japan, Hasegawa et al. [16] reported an angle of 52.3° (mean participant age, 39.4 years), and in the present study, the mean angle was 52 ± 10.0° for all ages and 50.9 ± 9.8° for those aged under 50 years (Table 2). Yeh et al. [15] reported that SS was greater in the 61–80-year age group than in the 41–60-year age group (31 ± 10 and 35 ± 9, respectively). The PI and SS obtained for this population appeared to be lower than those reported previously for Caucasian populations. In Japan, Hasegawa et al. [16] reported a mean age of 39.4 years and an angle of 40.8° based on a study involving healthy Japanese volunteers. In the present study, the mean of all age groups was 35.5 ± 9.0, while it was 38.0 ± 7.9 for Group 1 and 32.5 ± 10.7 for Group 4, showing a significant difference between these groups. The PI and SS results of this study are close to those reported previously [16,17]. It is suggested that the PI and SS of Asians, including Japanese people, may be lower than those reported for Caucasians (Table 2). Therefore, when obtaining the reference values of various parameters, comparisons should be made with those of Asians.

Yeh et al. [15] reported that the mean LL for all groups was 45 ± 15; 49 ± 12 for the 20–40 age group; 46 ± 14 for the 41–60 age group; and 40 ± 17 for the 61–80 age group. Moreover, the mean TK for all age groups was 33 ± 12, while it was 35 ± 10 for the 20–40 age group, 32 ± 13 for the 41–60 age group, and 31 ± 13 for the 61–80 age group. Hasegawa et al. [16] reported an LL of 40.4 and a TK of 41.5. In the present study, LL and TK were found to decrease gradually, as previously reported [14]. The overall average of the individuals in Group 1, comprising younger adults, and that of Groups 3 and 4, including older adults, were similar. However, the age-based grouping made it difficult to compare these participants (Table 3 and Table 4). Konishi et al. [17] found that degeneration and compensation in the sagittal plane of the spine–pelvis began in relatively flexible areas with a wide range of motion and gradually spread distally to the hip and lower extremities. Xu et al. [18] found that SS is the basis of lumbar kyphosis; smaller SS indicates smaller LL, but SS changes as the pelvis is anteriorly tilted and the spine degenerates. Therefore, SS is not suitable for guiding orthopedic strategies, and PI is the only morphological parameter that is strongly positively correlated with LL. LL changes may spread to the thoracic spine and pelvis. Yeh et al. [15] reported that PI in the 41–60 and 61–80 age groups was greater than that in the 20–40 age group, while PT increased with age. Regarding TK, a significant difference was only found between the groups aged 20–40 and 61~80. Konishi et al. [17] and Xu et al. [18] reported that LL changes occur first and are compensated by pelvis/TK; nonetheless, our study and that by Yeh et al. [15] suggested that pelvis/TK changes may occur first and be subsequently compensated by LL.

There are indications that spine/pelvis/hip surgery affects spinal alignment and pelvic morphology. Yeh et al. [15] suggested that there is a difference between Asians and Caucasians with regard to these morphological parameters. They recommended using Asian parameters as target values for correction when performing surgery on Asians. Notably, our study also suggested a difference between Japanese people and Caucasians. However, in younger patients, the sacroiliac joints compensate for TK loss, and in older patients, the sacroiliac joints lose motion, suggesting that the lumbar spine and the thoracic and lumbar vertebrae may compensate for the sacrum. Haffer et al. [19] and others have reported that hip arthroplasty alters spinal alignment. We hypothesized that in real clinical practice, pelvic changes in patients younger than 60 years are compensated for by the sacroiliac joint; thus, the implant that controls the movement of the sacroiliac joint should be removed when the sacroiliac joint is fixed, due to reasons such as pelvic ring fracture. In older adult patients, changes in the pelvis are compensated for by the spine; therefore, caution is necessary when performing THA after spinal fusion surgery, and in such patients, THA should be performed before spinal fusion surgery if possible. However, it is widely acknowledged [20,21,22] that post-spinal fusion patients experience more complications from THA compared to those without such a history. Consequently, Giai Via et al. [20] suggested that after spinal fusion surgery, patients should undergo a thorough preoperative examination, including careful implant and approach selection, as well as detailed intraoperative support and other surgical planning considerations. Conversely, sacroiliac joint fusion does not seem to influence changes in the pelvis or spine. Le Huec et al. [23] and Barry et al. [24] noted that the greater the PI, the greater the degree of posterior pelvic tilt. As Imai et al. [6] reported, there is a deep association between PI and a-SS, and a-SS is useful for estimating PI. Hence, as Łaziński et al. [25] reported, patient classification based on the mobility of the vertebral pelvis and sagittal spinal balance seems important for identifying these patients. It would be beneficial to investigate this association further to avoid additional radiography and to statistically predict spinal lesions based on preoperative factors. Therefore, it is important to measure a-SS before THA.

The present study had some limitations. We studied changes over time using age group parameters rather than studying the changes in the same individuals. Furthermore, it is necessary to study the changes in the pelvic and sagittal alignment of young patients undergoing THA using the same patient population and the differences between THA and non-THA patients. Additionally, Kim et al. [26] noted that the mobility of the pelvis, spine, and hip joints is interrelated before and after THA. It will also be necessary to look at the interrelationships in healthy individuals stratified by age group.

## 5. Conclusions

It is generally believed that degeneration begins in the highly mobile lumbar spine and is compensated for by changes in the pelvis and thoracic spine. However, in younger patients, the sacroiliac joint compensates for the loss of kyphosis in the thoracic spine. In older patients, the sacroiliac joint loses motion, suggesting the possibility of compensation by the lumbar and thoracic spine. If THA is to be performed, a-SS should be measured before surgery, considering the patient’s age.

In addition, when obtaining reference values for various parameters, it is advisable to use the data obtained from Asian patients as the reference values.

## Figures and Tables

**Table 1:** Various parameters by age group.

	Whole	<50	50–59	60–69	>70
	*n* = 340	*n* = 48	*n* = 97	*n* = 109	*n* = 76
APPA	−6.8 ± 9.3	−0.8 ± 6.9	−3.9 ± 8.8	−7.3 ± 7.9	−13.3 ± 9.4
SSx	35.5 ± 9.0	38.0 ± 7.9	36.9 ± 7.9	35.8 ± 8.9	32.5 ± 10.7
PTx	16.3 ± 7.3	12.9 ± 6.9	14.7 ± 7.4	16.1 ± 5.7	20.6 ± 8.1
PI	52.0 ± 10.0	50.9 ± 9.8	51.5 ± 10.4	52.5 ± 9.1	53.3 ± 10.9
a-SS	42.4 ± 10.4	38.9 ± 10.0	40.8 ± 9.7	43.6 ± 9.8	45.6 ± 11.5
a-PT	9.6 ± 7.8	12.1 ± 6.6	10.8 ± 8.0	8.9 ± 7.6	7.6 ± 9.0
TK	35.1 ± 12.0	39.6 ± 8.4	35.9 ± 8.6	33.5 ± 8.6	32.1 ± 9.8
LL	48.5 ± 12.1	53.8 ± 10.8	50.3 ± 11.6	47.6 ± 11.9	43.5 ± 14.0

**Table 2:** SS angles by age group.

Study	SS (°)					Difference (*p* < 0.05)
Yeh et al. [15]	All ages 33 ± 9	Group A 20–40 years 34 ± 9	Group B 41–60 years 35 ± 9	Group C 61–80 years 31 ± 10		B > C
Hasegawa et al. [16]		Average age 39.4 years 40.8				
This study	All ages 35.5 ± 9.0	Group 1 Under 50 years 38.0 ± 7.9	Group 2 50–59 years 36.9 ± 7.9	Group 3 60–69 years 35.8 ± 8.9	Group 4 Over 70 years 32.5 ± 10.7	1 > 4

**Table 3:** LL angles by age group.

Study	LL (°)					Difference (*p* < 0.05)
Yeh et al. [15]	All ages 45 ± 15	Group A 20–40 years 49 ± 12	Group B 41–60 years 45 ± 14	Group C 61–80 years 40 ± 15		C < B C < A
Hasegawa et al. [16]		Average age 39.4 years 40.4				
This study	All ages 48.5 ± 12.1	Group 1 Under 50 years 53.8 ± 10.8	Group 2 50–59 years 50.3 ± 11.6	Group 3 60–69 years 47.6 ± 11.9	Group 4 Over 70 years 43.5 ± 14.0	1 > 3 1 > 4 2 > 4

**Table 4:** TK angles by age group.

Study	TK (°)					Difference (*p* < 0.05)
Yeh et al. [15]	All ages 33 ± 12	Group A 20–40 years 35 ± 10	Group B 41–60 years 32 ± 13	Group C 61–80 years 31 ± 13		C < A
Hasegawa et al. [16]		Average age 39.4 years 41.5				
This study	All ages 35.1 ± 12.0	Group 1 Under 50 years 39.6 ± 8.4	Group 2 50–59 years 35.9 ± 8.6	Group 3 60–69 years 33.5 ± 8.6	Group 4 Over 70 years 32.1 ± 9.8	1 > 2 1 > 3 1 > 4

## Data Availability

The datasets used and/or analyzed during the current study are available from the corresponding author upon reasonable request.
